# Reduced serum adiponectin and exercise predict depression in chronic heart failure patients and affect depressive-like behaviors in a mouse model

**DOI:** 10.3389/fphys.2026.1835186

**Published:** 2026-09-22

**Authors:** Hui Yang, Qian Du, Fang Zeng, Biao Li

**Affiliations:** Department of Cardiology, Guangzhou Red Cross Hospital of Jinan University, Guangzhou, China

**Keywords:** adiponectin, depression, exercise, heart failure, prevention

## Abstract

**Background:**

Chronic heart failure (CHF) is frequently comorbid with depression and anxiety. Currently, we aim to investigate the association between serum adiponectin levels and depression among patients with CHF.

**Methods:**

A total of 119 patients with HF in a tertiary hospital in southern China were enrolled. All patients completed the Patient Health Questionnaire-9 (PHQ-9) upon discharge. Serum levels of adiponectin, ghrelin, leptin A, and BDNF were measured. On the basis of PHQ-9 scores, HF patients were divided into non-Dep, mild-Dep, moderate-Dep, and severe-Dep groups. Differences in the levels of cardiac markers and serum mediators between the non/mild-Dep group and the moderate/severe-Dep group were analyzed using weighted univariate and multivariate logistic regression models. An HF C57BL/6 mouse model of depression was established. AdipoRon, an orally active adiponectin receptor agonist, was administered to the mouse model.

**Results:**

A total of 119 patients with CHF were categorized into non-Dep (n=35), mild-Dep (n=46), moderate-Dep (n=20), and severe-Dep groups (n=18). Compared with those in the non-Dep group, adiponectin levels in the Dep group were significantly lower and correlated with increased depression severity in HF patients. Weighted univariate and multivariate regression analyses indicated that adiponectin (OR = 0.719; 95% CI: 0.531–0.975) and exercise duration (OR = 0.655; 95% CI: 0.489–0.877) were potential predictors of CHF comorbid with depression. Spearman correlation analysis revealed a positive correlation between adiponectin levels and exercise frequency/duration, whereas negative correlations were found between depression scores and exercise frequency/duration. Sensitivity analyses with stepwise exclusion of high-risk subgroups (diabetes, systemic inflammation, and severe HF) revealed a significant association between exercise duration and depression. In a depression model in MI mice, serum adiponectin levels were decreased. Compared with single-treatment approaches, the combination of adiponectin receptor agonists and exercise intervention effectively improved depressive-like behaviors in mice and regulated related biomarker levels, with superior effects.

**Conclusion:**

Reduced serum adiponectin levels and exercise activity were associated with increased odds of having CHF comorbid with depression. Activation of the adiponectin/AdipoR1 pathway combined with exercise intervention has potential benefits for the prevention of depression in patients with HF.

## 1 Introduction

Heart failure (HF) is a clinical syndrome caused by impaired cardiac systolic and/or diastolic function and is characterized by venous congestion and arterial hypoperfusion (Fudim et al., 2022). Studies have shown that 10% to 16% of HF patients develop cachexia (Palus et al., 2011). The prevalence of psychological disorders such as anxiety and depression is even higher. Among global HF patients meeting the diagnostic criteria for moderate to severe depression, the prevalence rates of anxiety and depression reach 41.9% and 28.1%, respectively (Moradi et al., 2022). This proportion is significantly higher than the previously reported prevalence (21.5%) in 2006 (Rutledge et al., 2006). Depression has been confirmed to be an independent risk factor for poor HF prognosis. Symptoms of depression, such as anxiety, irritability, and insomnia, reduce patients’ quality of life and severely impact the prognosis of HF patients (Shimizu et al., 2014). A nationwide cohort study revealed that HF combined with depression markedly increased the risk of suicide among patients. The suicide risk peaks within three months after the diagnosis of HF (Crump et al., 2022). There is a bidirectional association between HF and anxiety or depression. HF can induce or exacerbate psychological disorders through pathophysiological changes, whereas anxiety/depression can further worsen the clinical outcomes of HF.

The hypothalamic–pituitary–adrenal (HPA) axis, as the body’s stress sensor, has been confirmed by studies to be a key factor in triggering HF combined with depression (Troubat et al., 2021). The effect of the HPA axis is stronger in elderly depressive patients than in nonelderly depressive patients. When patients are in a prolonged negative emotional state, their cortisol secretion levels exhibit more pronounced hyperactivity. Overactivity of the HPA system leads to excessive secretion and release of various hormones, such as adrenaline and angiotensin II, thereby increasing myocardial oxygen consumption, causing elevated blood pressure, severe water and sodium retention, and further exacerbating cardiac preload (Stetler and Miller, 2011). Additionally, patients with CHF experience restricted cardiac ejection function (Xu et al., 2022) and insufficient oxygen levels in tissue cells (Poole et al., 2012). Thus, their bodies remain in a prolonged state of stress. Hyperactivity of the HPA axis and sympathetic nerves, along with excessively high levels of hormones (e.g., norepinephrine and adrenaline), can stimulate the central regulatory functions of the brain, generating or worsening negative emotions such as depression (Menke, 2024). The activation of inflammatory pathways is also an adaptive response to HF stress, in which the body exerts cardioprotective effects by promoting cardiomyocyte hypertrophy and inhibiting apoptosis. When the blood–brain barrier (BBB) is compromised, an overactivated inflammatory response may trigger neuroinflammation and brain dysfunction (Cacciotti et al., 2023).

Adiponectin is a hormone that is primarily secreted by white adipose tissue and is expressed at low levels in osteoblasts, skeletal muscle, and myocardial cells. Adiponectin has multiple functions, including regulating energy metabolism, anti-inflammatory effects, antioxidant stress, anti-apoptosis, and maintaining vascular homeostasis (Chen et al., 2003; Ohashi et al., 2014). Since its discovery in 1995 (Scherer et al., 1995), research on the functions of adiponectin has expanded from peripheral metabolic regulation to the central nervous system. Although adiponectin is produced mainly peripherally, it can cross the BBB (Liu et al., 2024) and bind to specific receptors (including adipoR1 and adipoR2) in the hypothalamus, brainstem, hippocampus, and cortex (Gianopoulos et al., 2025). These brain regions (including the amygdala, hypothalamus, cortex, and hippocampus) are the key areas involved in the pathology of anxiety and depression (Espasandín et al., 2022). Experimental evidence confirms that adiponectin has clear central protective effects. Intracerebroventricular injection of adiponectin can reduce neuroinflammation, promote hippocampal neural stem cell proliferation, exhibit antianxiety and antidepressant effects, and accelerate fear memory extinction (Zhang et al., 2017). Adiponectin also regulates central neuropeptide Y neurons (Hoyda et al., 2009) or reduces peripheral renal sympathetic nerve activity (Tanida et al., 2007), thereby lowering peripheral blood pressure. Additionally, adiponectin can inhibit gastric emptying to participate in peripheral satiety regulation, which may lead to central anorexigenic effects in rodents (Idrizaj et al., 2024).

On the basis of the current findings, we propose that serum adiponectin is associated with the development of depression in patients with CHF. We analyzed the clinical data of CHF patients with comorbid depression to identify independent risk factors that may induce depressive mood. Additionally, we established a mouse model of CHF combined with depression. Following treatment with the adiponectin receptor agonist AdipoRon and exercise intervention, we further clarified the therapeutic effects of adiponectin and exercise on CHF-related depression. It is hoped that this study will provide new treatment strategies for patients with HF progressing to depression.

## 2 Materials and methods

### 2.1 Clinical samples

This was a retrospective study. From June 2023 to June 2024, 119 middle-aged and elderly CHF patients hospitalized at Guangzhou Red Cross Hospital of Jinan University were selected. Additionally, 40 non-CHF samples were included as a control group. This study was approved by the Ethics Committee of Guangzhou Red Cross Hospital of Jinan University (No. LL-2023-016-01). All participants provided written informed consent. The Patient Health Questionnaire-9 (PHQ-9) (a Chinese version) was used to assess depression scores in the patients (Wang et al., 2014). A Cronbach’s alpha of 0.86 confirmed the internal consistency reliability of this scale. The area under the curve of the ROC curve was 0.92 (95% confidence interval: 0.86–0.97). A cutoff score of 7 or higher on the PHQ-9 had a sensitivity of 0.86 and a specificity of 0.86. In this study, mild depression (5–9 points), moderate depression (10–14 points), and severe depression (>15 points) were defined on the basis of the scores. All clinical and laboratory measurements were taken at a single time point (enrollment).

The following is the definition of the CHF. (1) HF with reduced ejection fraction (HFrEF): Left ventricular ejection fraction (LVEF) <40%, accompanied by typical signs of HF (such as elevated jugular venous pressure ≥8 cm H_2_O, peripheral edema) and symptoms corresponding to NYHA functional class II-IV; (2) HF with preserved ejection fraction (HFpEF): LVEF ≥50%, combined with HF symptoms and signs, B-type natriuretic peptide (BNP) >35 pg/mL or NT-proBNP >125 pg/mL, and significant cardiac structural/functional abnormalities; (3) HF with mid-range ejection fraction (HFmrEF): LVEF 40%-49%, requiring simultaneous fulfillment of the biomarker and structural abnormality criteria for HFpEF as described above.

The inclusion criteria for patients were as follows: (1) had clinically confirmed chronic HF with a prognosis >6 months; (2) met the diagnostic criteria for depression (Wang et al., 2014): had a PHQ-9 depression score >4; (3) did not currently receive any antidepressant treatment; and (3) voluntarily signed informed consent, fully understood the study protocol, and were committed to complete follow-up. All the subjects met all the above conditions. Individuals were excluded if they 1) had severe liver or kidney impairment (eGFR <30 mL/min/1.73 m² or need for dialysis), 2) had autoimmune or infectious disorders capable of influencing metabolic biomarkers, or 3) had incomplete clinical and laboratory information necessary for the analysis.

### 2.2 Experimental animals

Male C57BL/6 mice (8 weeks of age, weighing 21–25 g) were employed in this study. All the animals were supplied by Guangdong Zhiyuan Biopharmaceutical Technology Co., Ltd., China. The mice were housed under standardized environmental conditions with a temperature of 23 ± 2 °C, a relative humidity of 50 ± 5%, and a 12 h light–dark cycle, with ad libitum access to food and water throughout the experiment. All animal procedures were performed in compliance with the National Institutes of Health Guide for the Care and Use of Laboratory Animals and were approved by the Ethics Committee of Guangzhou Red Cross Hospital (Approval No. LL-2023-016-01).

### 2.3 Animal model establishment

A mouse model of HF was established by left anterior descending coronary artery ligation-induced myocardial infarction (MI) (n = 15). The mice were anesthetized by intraperitoneal injection of 1% sodium pentobarbital (40 mg·kg^^-^¹^; #P5178; Sigma–Aldrich). After the animals exhibited loss of the corneal reflex and decreased muscle tone, they were placed in a supine position on a rodent board and intubated for connection to a ventilator. A left thoracotomy was performed between the 3rd and 4th intercostal spaces, followed by layer-by-layer dissection of the subcutaneous tissue. After the chest was opened, the heart was exposed, and the left anterior descending coronary artery was ligated with a nontraumatic suture needle below the left auricle. Pallor of the anterior descending artery-supplied area was visually confirmed. The thoracic cavity was then compressed to expel the air and closed layer by layer. The sham surgery group (n=15) underwent the same procedure but without artery ligation. Penicillin (160 U·kg^^-^¹^·d^^-^¹^) was administered subcutaneously for 3 days to prevent infection.

To establish a depression model in MI mice, chronic unpredictable mild stress (CUMS) was initiated on the 8th day after left anterior descending coronary artery ligation surgery. The CUMS initiation day was set as day 0. The CUMS procedure lasted for 14 consecutive days. The procedures included fasting (24 h with free access to water), water deprivation (24 h with free access to food), circadian rhythm disruption (24 h light/dark cycle inversion), damp bedding (24 h with bedding containing approximately 30% moisture), cage tilt (45° maintained for 24 h), physical restraint (fixed in a transparent cylindrical restrictor for 2 h), and mechanical stimulation (clamping the tail root for 1 min with force sufficient to avoid skin damage). Each week followed a predetermined sequence to ensure that each stimulus was applied once per week, with no repetition of the same stimulus on consecutive days.

From day 14, the MI mice received either an injection of AdipoRon (an adiponectin receptor agonist), treadmill exercise (forced running at 10 m/min for 20 min/day, 5 days/week), or an injection of AdipoRon combined with treadmill exercise. Treatment group: (1) AdipoRon group: AdipoRon (HY-15848; MedChemExpress, USA) was dissolved in DMSO and then diluted in PEG-300 and Tween-80 to yield a final dosage of 20 mg/kg for daily intraperitoneal (i.p.) injection (Zhou et al., 2023; Nie et al., 2025). (2) Run group: The Run group was subjected to moderate-intensity training on a mouse treadmill (Liu et al., 2024). (3) AdipoRon+Run group: Each group consisted of 8 mice, and the mice were euthanized immediately on the 28th day post-surgery. Behavioral tests were performed at three time points: day 0 (before surgery), day 14, and day 28. Serum samples were collected on day 28.

### 2.4 Behavioral assessment

Sucrose Preference Test: Mice were housed individually. A 1% (w/v) sucrose solution was prepared. During the adaptation period, each cage was provided with one bottle of sucrose solution and one bottle of purified water, allowing free access. After 24 hours, the positions of the bottles were swapped to prevent positional preference. After a 24-hour fasting and water deprivation period following the 3-day adaptation, the test began. Preweighed sucrose solution and purified water bottles were placed in the cages. After 6 hours, the bottles were removed and weighed. Sucrose solution and pure water consumption were recorded, and the sucrose preference index was calculated. Sucrose preference index = (sucrose solution consumption/(sucrose solution consumption + pure water consumption)) × 100% (Zhao et al., 2025).

Tail Suspension Test: The proximal mouse tail (1 cm) was attached with medical tape to a suspension holder that was positioned 50 cm vertically above the experimental platform surface. A rigid plastic tube was placed over the distal tail to prevent climbing or tail entanglement with supports. The entire process was recorded continuously for 6 minutes using a high-definition camera, and the immobility time during this period was measured (Asim et al., 2024).

### 2.5 ELISA

After centrifugation, serum samples from each patient were collected and stored at -80 °C. The expression levels of CRP, adiponectin, ghrelin, BDNF, leptin, and NT-proBNP, including CRP (#ml300545), adiponectin (#ab314604, Abcam), ghrelin (#ml060543), BDNF (#ml105982), leptin (#ml028544), and NT-proBNP (#ml061452) in clinical samples, were detected using commercial ELISA kits. The levels of adiponectin and NT-proBNP in mouse serum were measured using adiponectin (#ml107260) and NT-proBNP (#ml001918) kits. With the exception of the human adiponectin kit, all the other kits were purchased from Enzyme-Linked Biotechnology (Hangzhou, China).

All the ELISAs were performed in a sandwich format and strictly followed the manufacturers’ instructions. Measurements were performed in triplicate. Briefly, serum samples were diluted according to the manufacturer’s recommended dilution folds (human samples: 1:500 for CRP, 1:100 for adiponectin, 1:50 for ghrelin, 1:80 for BDNF, 1:200 for leptin, and 1:20 for NT-proBNP; mouse samples: 1:200 for adiponectin and 1:10 for NT-proBNP). In brief, 100 µL of diluted sample or standard was added to each well and incubated for 2 h at room temperature. After three washes with 300 µL of washing buffer, 100 µL of biotinylated detection antibody was added, and the samples were incubated for 1 h at room temperature. Following another washing step, 100 µL of horseradish peroxidase-streptavidin was added and incubated for 30 min in the dark. After a final wash, 100 µL of tetramethylbenzidine substrate was added and incubated for 15 min in the dark. The reaction was stopped with 50 µL of H_2_SO_4_, and the optical density was read at 450 nm using a microplate reader (Multiskan FC, Thermo Fisher Scientific).

### 2.6 Statistical analysis

All the statistical analyses were performed using SPSS 24.0, and the figures were generated with GraphPad Prism 10.5. Normally distributed continuous variables are expressed as the mean ± standard deviation (SD) and were compared by independent-samples t tests; nonnormally distributed variables are presented as medians (interquartile ranges) and were analyzed using the Mann–Whitney U test. Categorical variables are shown as numbers (%) and were examined by the chi-square (χ²) test. Pearson correlation analysis was used to assess linear correlations between exercise duration, PHQ-9 scores, and serum levels of BDNF, adiponectin, ghrelin, leptin, and CRP. P < 0.05 was considered to indicate statistical significance. To evaluate the robustness of our primary findings and to address potential confounding by key comorbidities, we performed sensitivity analyses. Specifically, we sequentially excluded patients with (1) diabetes mellitus; (2) elevated systemic inflammation (high-sensitivity CRP > 0.3 mg/dL, according to the AHA/CDC cutoff for high cardiovascular risk) (Albini et al., 2023); and (3) severe HF (NT-proBNP above the upper tertile of the cohort distribution). In each subsample, a fully adjusted multivariable logistic regression model was constructed to examine the stability of the core predictors. The results from the sensitivity analyses were visualized using a forest plot to facilitate comparison of effect sizes (odds ratios) across the main and sensitivity cohorts.

## 3 Results

### 3.1 Clinical characteristics and metabolic parameters

This study included a total of 119 elderly patients with CHF, including 80 males and 39 females. Among them, 35 had no depressive state, and 84 had depressive states (including 46 with mild depression, 20 with moderate depression, and 18 with severe depression). In the control group, 40 non-CHF individuals—27 males and 13 females—were included. Among them, 24 individuals had no depressive symptoms, and 16 individuals had a depressive state (including 11 with mild depression, 4 with moderate depression, and 1 with severe depression). The baseline clinical characteristics of the two groups are detailed in **Table 1**. Comparative analysis of baseline characteristics revealed no statistically significant differences between the two groups in terms of demographic/lifestyle indicators such as age, body mass index (BMI), sex, smoking history, or living alone (P > 0.05). However, the incidence of depressive symptoms in the CHF group was significantly greater than that in the non-CHF group (P < 0.05). Additionally, in terms of biomarker levels, compared with the non-CHF group, the CHF group had significantly higher levels of NT-proBNP, CRP, and leptin (P < 0.05). Conversely, the CHF group exhibited significantly lower levels of BDNF, adiponectin, and ghrelin than the non-CHF group did (P < 0.05).

**Table 1:** Baseline clinical characteristics and metabolic parameters of patients with chronic heart failure.

Parameter	Non-CHF (n=40)	CHF (n=119)
Age (years)	66.68 ± 9.08	67.58 ± 12.24
Female sex (%)	13 (32.50%)	39 (32.77%)
BMI (kg/m^2^)	25.22 ± 3.20	24.77 ± 3.47
NYHA functional class		
I		3
II		44
III		52
IV		20
Alcohol Consumers	20 (50%)	53 (44.54%)
Smoke	19 (47.50%)	45 (37.82%)
Living without partner	19 (47.50%)	41 (34.45%)
Hypertension	16 (40%)	49 (41.18%)
Diabetes mellitus	10 (25%)	33 (27.73%)
Depressive symptom*	16 (40%)	84 (70.59%)
Mild depression	11 (27.50%)	46 (38.66%)
Moderate depression	4 (10%)	20 (16.81%)
Severe depression*	1 (2.5%)	18 (15.13%)
Exercise times/week*	3.90 ± 1.80	2.29 ± 1.64
Exercise time*	9.40 ± 6.30	5.0 (2.0,9.0)
NT-proBNP (pg/mL)*	31.97 (20.78,41.13)	2125 (466.0,4339.0)
High-sensitive CRP (mg/dL)*	0.12 ± 0.05	0.37 (0.25,0.75)
BDNF (ng/ml)*	25.44 ± 9.53	16.10 (11.52,22.60)
Adiponectin (µg/ml)*	11.38 ± 4.74	6.90 (4.20,10.50)
Ghrelin (pg/ml)*	881.50 ± 411.30	390.60 (277.30,566.50)
Leptin (μg/L)*	12.92 ± 8.53	30.70 (19.50,49.60)

CHF, chronic heart failure; BDNF, brain-derived neurotrophic factor; BMI, body mass index; CRP, C-reactive protein; NYHA, New York Heart Association. The values are presented as the mean ± standard deviation (SD) or percentages. *indicates a significant difference according to ANOVA (p < 0.05). Variables that did not follow a normal distribution (as assessed by the Shapiro–Wilk test) are presented as medians (Q1, Q3). For normally distributed variables, we presented the data as the mean ± SD.

According to the level of depression, the control group and HF patients were divided into the normal control group (Control group), depression group (Dep group), CHF group, and CHF+Dep group. The demographic and clinical parameters are summarized in **Table 2**. Statistical analysis revealed great differences among groups in terms of diabetes incidence, weekly exercise frequency, exercise duration, NT-proBNP level, and high-sensitivity CRP, BDNF, adiponectin, ghrelin, and leptin levels. Specifically, compared with the Dep group and CHF group, the CHF+Dep group had a markedly greater incidence of diabetes. This comorbidity group also exhibited higher levels of NT-proBNP and hs-CRP (**Figures 1A, B**). Conversely, the concentrations of BDNF, ghrelin, and leptin in the CHF+Dep group were significantly lower than those in the Dep group. Both the CHF group and the CHF+Dep group had lower adiponectin levels than the control group did (**Figures 1C–F**). In terms of exercise, compared with the CHF group and Dep group, the CHF+Dep group had fewer weekly exercise sessions and shorter exercise durations (**Table 2**).

**Table 2:** Baseline clinical characteristics and metabolic parameters of patients with chronic heart failure and depression.

Parameter	Controls (n=24)	Dep (n=16)	CHF (n=35)	CHF+Dep (n=84)
Age (years)	66.67 ± 7.94	66.69 ± 10.85	65.17 ± 13.11	68.58 ± 11.80
Female sex (%)	10 (41.67%)	3 (18.75%)	13 (37.14%)	26 (30.95%)
BMI (kg/m^2^)	24.94 ± 3.46	25.65 ± 2.83	25.20 ± 3.91	24.59 ± 3.276
Alcohol Consumers	12 (50%)	8 (50%)	14 (40%)	39 (46.43%)
Smoke	11 (45.83%)	8 (50%)	17 (48.57%)	28 (33.33%)
Living without a partner	10 (41.67%)	9 (56.25%)	12 (34.29%)	29 (34.52%)
Hypertension	10 (41.67%)	6 (37.5%)	11 (31.43%)	38 (45.24%)
Diabetes mellitus*	6 (25%)	4 (25%)	5 (14.29%)	28 (33.33%)
Exercise times/week*	4.04 ± 1.49	3.688 ± 2.21	3.29 ± 1.76	1.88 ± 1.40
Exercise time*	9.52 ± 6.31	9.219 ± 6.49	8.83 ± 5.40	4.80 (2,5.75)
NT-proBNP (pg/mL)*	28.50 (20.85,37.95)	30.37 ± 20.43	521 (245,2640)	2460 (4231, 5093)
High-sensitive CRP (mg/dL)*	0.12 ± 0.05	0.13 ± 0.05	0.29 (0.24,0.58)	0.40 (0.26,0.85)
BDNF (ng/ml)*	26.29 ± 11.13	24.16 ± 6.59	19.02 ± 6.96	16.37 ± 6.68
Adiponectin (µg/ml)*	12.57 ± 4.31	9.60 ± 4.92	8.52 ± 4.10	6.75 (3.73,9.98)
Ghrelin (pg/ml)*	913.9 ± 375.8	832.9 ± 468.0	491.8 ± 243.3	416.3 ± 244.6
Leptin (μg/L)*	12.73 ± 7.57	13.19 ± 10.06	37.47 ± 20.40	33.27 ± 20.40

Dep, depressive symptoms; CHF, chronic heart failure; BDNF, brain-derived neurotrophic factor; BMI, body mass index; CRP, C-reactive protein. The values are presented as the means ± standard deviations or percentages. *indicates a significant difference according to the ANOVA test (p < 0.05). Variables that did not follow a normal distribution (as assessed by the Shapiro–Wilk test) are presented as medians (Q1, Q3), whereas normally distributed variables are presented as the mean ± SD.

**Figure 1:**
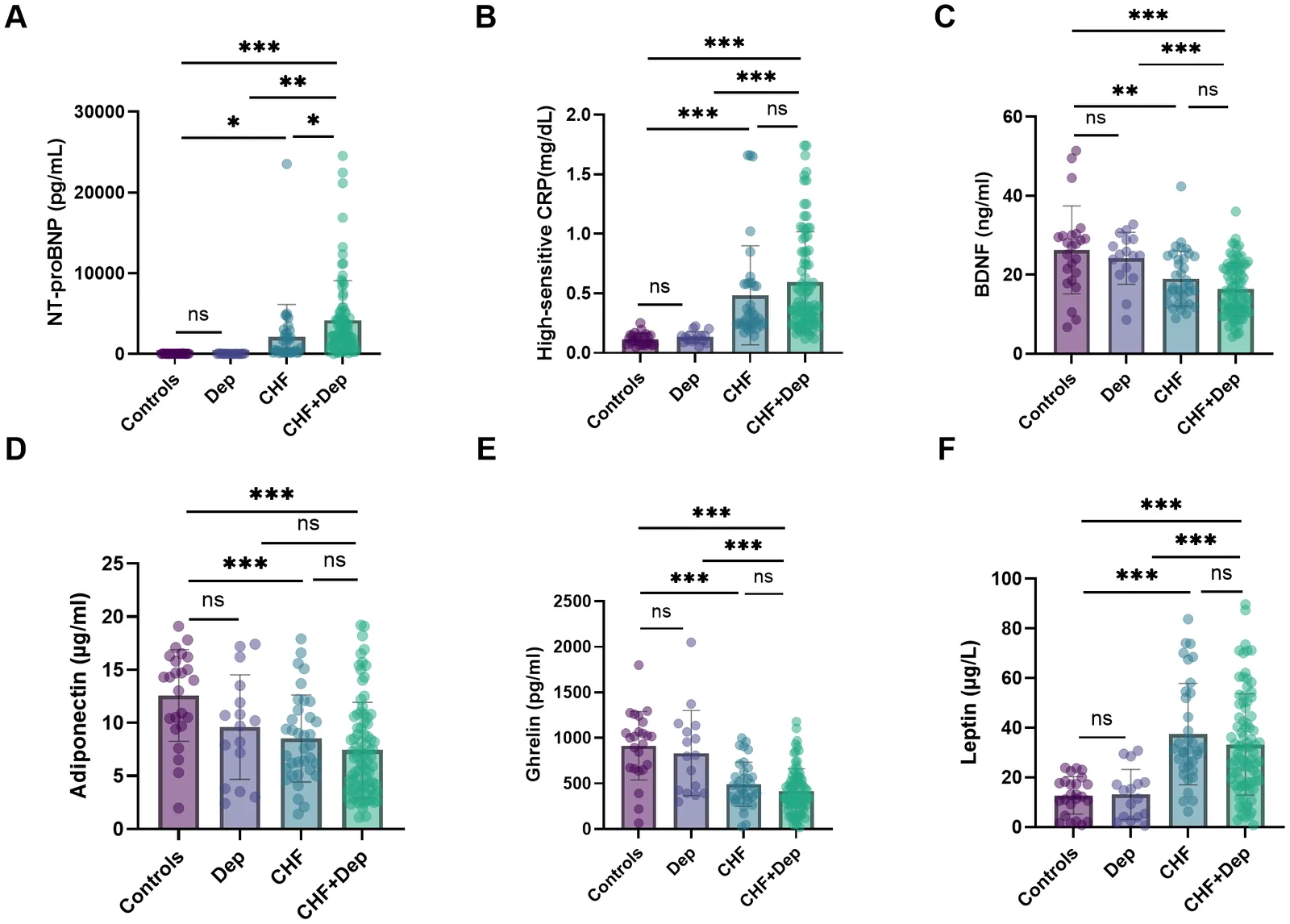
Adiponectin was downregulated in patients with chronic heart failure and depression. Comparisons of **(A)** NT-proBNP, **(B)** hs-CRP, **(C)** BDNF, **(D)** ghrelin, **(E)** leptin, and **(F)** adiponectin levels among the control, CHF, Dep, and CHF+Dep groups. *P<0.05, **P<0.01, ***P<0.001, ns P>0.05.

To further explore the differences between groups, we compared the serum levels of NT-proBNP, high-sensitivity CRP, BDNF, ghrelin, leptin, and adiponectin between the control group and the CHF group. Serum NT-proBNP, high-sensitivity CRP, and leptin levels were significantly higher in CHF patients than in non-CHF patients, whereas BDNF, ghrelin, and adiponectin levels were clearly lower in the CHF group (**Figures 2A–F**). Furthermore, CHF patients were categorized into mild-Dep, moderate-Dep, and severe-Dep groups on the basis of the PHQ-9 depression score. Subgroup analysis revealed that NT-proBNP and high-sensitivity CRP levels increased with increasing severity of depression (**Figures 2G, H**). Conversely, BDNF, ghrelin, and adiponectin levels decreased as depression severity increased (**Figures 2I–K**).

**Figure 2:**
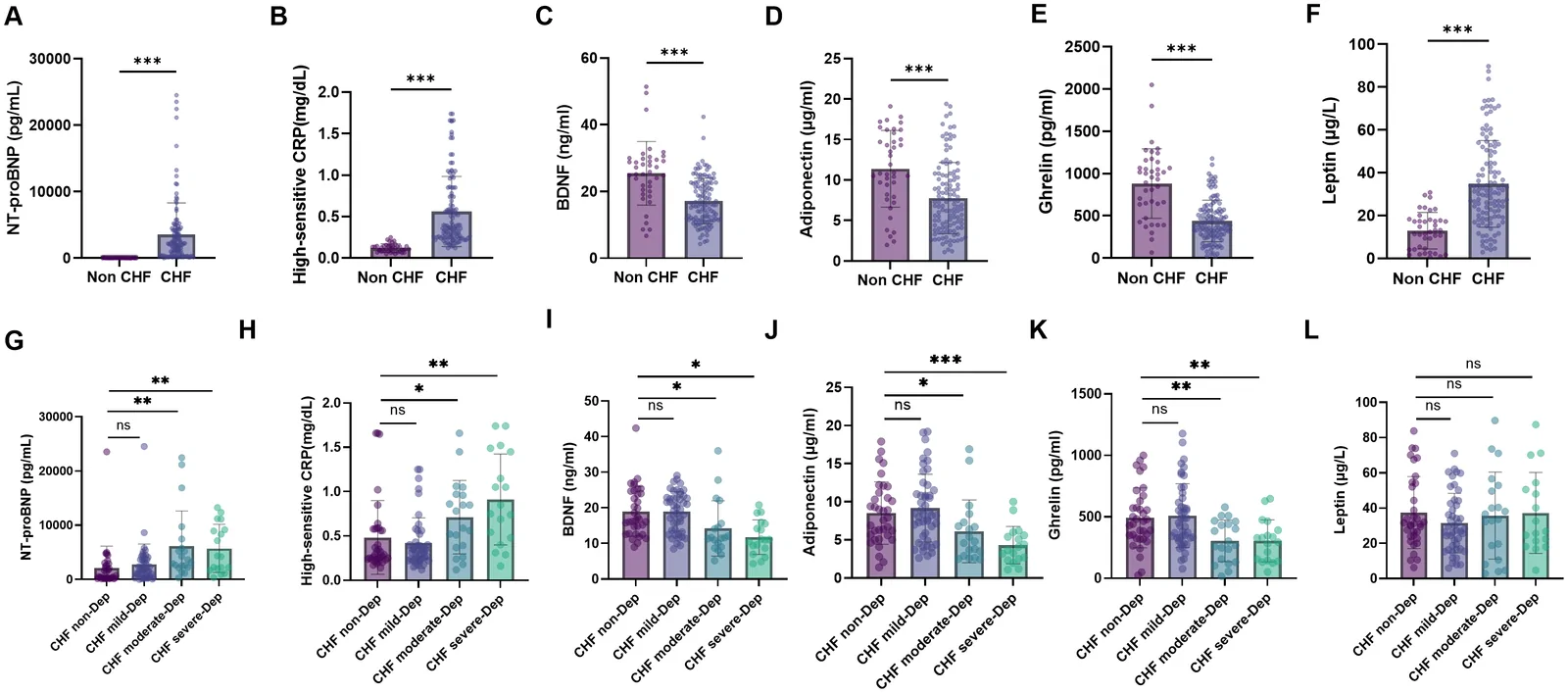
Downregulation of Adiponectin Levels in Heart Failure Patients. Analyze the differences in the levels of NT-proBNP **(A)**, high-sensitivity CRP **(B)**, BDNF **(C)**, adiponectin **(D)**, ghrelin **(E)**, and leptin **(F)** between the CHF and non-CHF groups. The differences in the levels of NT-proBNP **(G)**, high-sensitivity CRP **(H)**, BDNF **(I)**, adiponectin **(J)**, ghrelin **(K)**, and leptin **(L)** among the CHF non-Dep, CHF mild-Dep, CHF moderate-Dep, and CHF severe-Dep groups were analyzed. *P<0.05, **P<0.01, ***P<0.001, ns P>0.05.

### 3.2 Analysis of risk factors for depression

Through univariate and multivariate logistic regression model analyses, we identified key risk factors for depression among CHF patients (**Tables 3**, **4**). Univariate logistic regression analysis revealed that diabetes mellitus, exercise duration/week, exercise duration, and NT-proBNP, high-sensitivity CRP, BDNF, adiponectin, and ghrelin levels were significantly associated with the risk of depression. Further multivariate regression analysis confirmed that the core risk factors for depression in CHF patients included comorbid diabetes mellitus, shorter exercise time, higher levels of high-sensitivity CRP, and decreased levels of BDNF and adiponectin.

**Table 3:** Univariate logistic regression analysis to identify the risk factors for depression in patients with chronic heart failure.

Parameter	ß	SE of ß	OR	95% CI	*p* value
Age (years)	0.003	0.02	1.003	0.965-1.042	0.877
Female sex	-0.796	0.53	0.451	0.16-1.275	0.133
BMI (kg/m^2^)	-0.109	0.068	0.897	0.785-1.024	0.106
Alcohol Consumers	-0.022	0.479	0.978	0.383-2.50	0.963
Smoke	-0.841	0.492	0.431	0.164-1.132	0.088
Living without partner	0.439	0.483	1.552	0.602-3.998	0.363
Hypertension	0.569	0.489	1.766	0.677-4.605	0.245
Diabetes mellitus	1.253	0.589	3.5	1.104-11.094	0.033
Exercise times/week	-0.837	0.236	0.433	0.272-0.688	<0.001
Exercise time	-0.303	0.079	0.738	0.633-0.862	<0.001
NT-proBNP (pg/mL)	0	0	1	1.000-1.000	0.007
High-sensitive CRP(mg/dL)	1.725	0.625	5.61	1.649-19.083	0.006
BDNF (ng/ml)	-0.139	0.044	0.87	0.798-0.948	0.002
Adiponectin (µg/ml)	-0.244	0.077	0.784	0.674-0.911	0.001
Ghrelin (pg/ml)	-0.005	0.001	0.995	0.993-0.998	0.001
Leptin (μg/L)	-0.004	0.011	0.996	0.975-1.017	0.704

*indicates a statistically significant difference, p < 0.05; ß, regression coefficient; CI, confidence interval for odds ratio; OR, odds ratio; SE, standard error; BDNF, brain-derived neurotrophic factor; BMI, body mass index; CRP, C-reactive protein.

**Table 4:** Multivariate logistic regression analysis to identify the risk factors for depression in patients with chronic heart failure.

Parameter	ß	SE of ß	OR	95% CI	*p* value
Diabetes mellitus	3.563	1.478	35.261	1.946-638.836	0.016
Exercise time	-0.424	0.149	0.655	0.489-0.877	0.004
High-sensitive CRP (mg/dL)	2.012	0.996	7.479	1.061-52.722	0.043
BDNF (ng/ml)	-0.151	0.056	0.86	0.770-0.960	0.007
Adiponectin (µg/ml)	-0.329	0.155	0.719	0.531-0.975	0.034

*indicates a statistically significant difference, p < 0.05; ß, regression coefficient; CI, confidence interval for odds ratio; OR, odds ratio; SE, standard error; BDNF, brain-derived neurotrophic factor; CRP, C-reactive protein.

To examine whether these associations were biased by residual confounding from HF severity, inflammation, or diabetes, we performed sensitivity analyses with stepwise exclusion of high-risk subgroups. As shown in the forest plot (**Figure 3**), the inverse association between exercise duration and depression remained consistently significant and stable across all sensitivity scenarios (CHF cohort: OR = 0.655, 95% CI: 0.489–0.877; excluding diabetes: OR = 0.847, 95% CI: 0.762–0.941; excluding high CRP: OR = 0.772, 95% CI: 0.641–0.930; excluding severe HF: OR = 0.820, 95% CI: 0.726–0.925). In contrast, the effects of BDNF and adiponectin were substantially attenuated and became nonsignificant after these high-risk groups were excluded (all 95% CIs crossed 1.0). Similarly, the extremely large effect sizes of diabetes (OR = 35.26 in the main analysis) and hs-CRP (OR = 7.48) decreased to near-unity and were no longer statistically significant in the sensitivity subsets. These sensitivity analyses collectively demonstrate that exercise duration is the most robust and generalizable predictor of depression in CHF patients, whereas the predictive value of the evaluated biomarkers is largely dependent on the presence of severe metabolic or inflammatory derangements.

**Figure 3:**
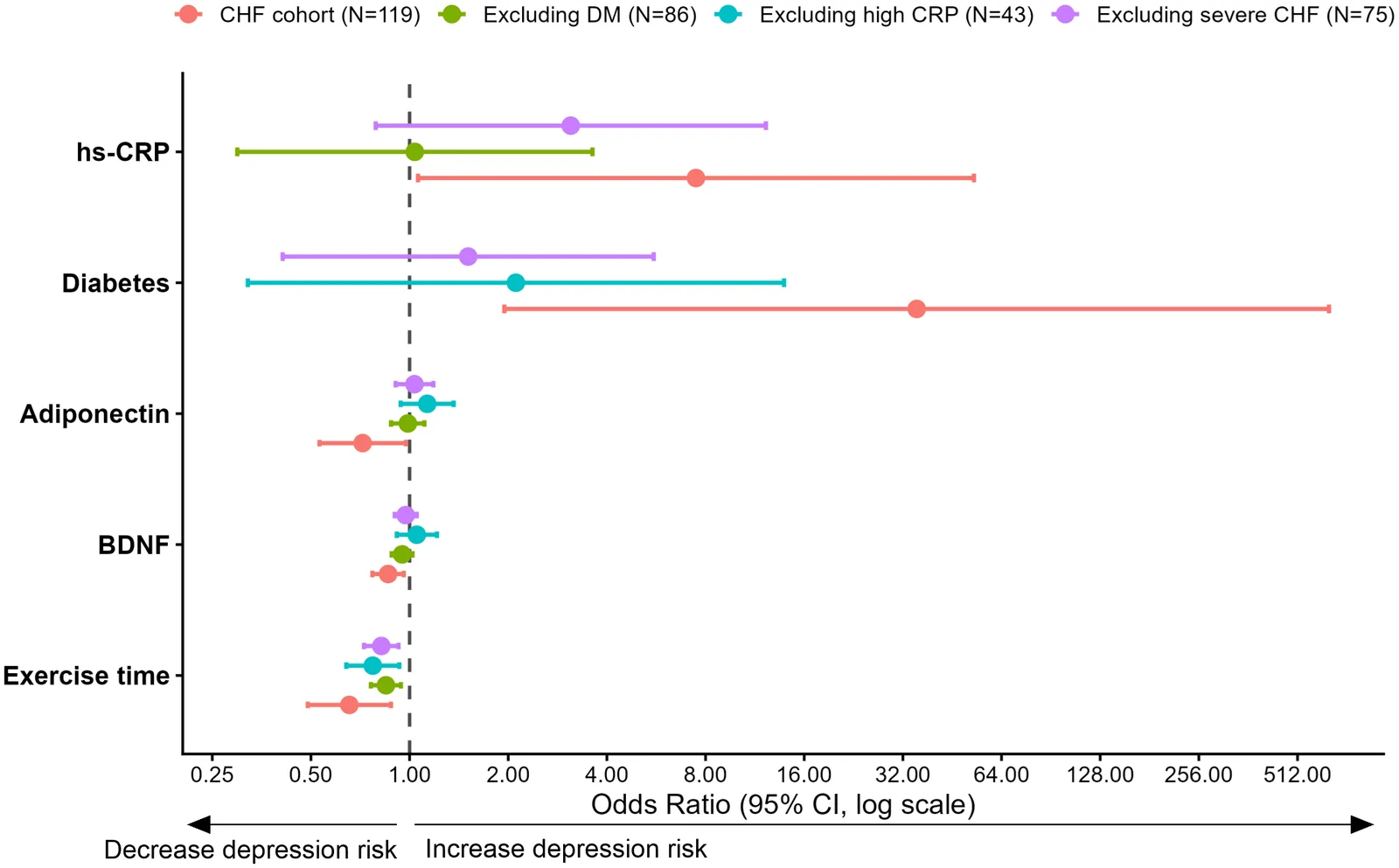
Forest plot visualizing the causal effects of factors on depression in heart failure patients.

To explore the association between exercise frequency and depression severity, we retrospectively surveyed patients with depression (including both those with and those without comorbid CHF) regarding their self-reported exercise frequency over the past 12 months. A total of 100 patients with depression were included. Patients were divided into three groups on the basis of exercise frequency: once or less per week (≤1/week, also indicating nonstructured or erratic exercise; n=30), two or three times per week (2–3/week; n=53), and more than three times per week (>3/week; n=17). We found that exercise frequency had a significant moderating effect on depression scores and multiple biomarkers. The PHQ-9 depression scores in the high-frequency exercise group (>3/week) were markedly lower than those in the low-frequency group (≤1/week) and the medium-frequency group (2–3/week; **Figure 4A**). Moreover, adiponectin levels were lower in the >3/week group than in the 2–3/week group (**Figure 4B**). Notably, BDNF levels tended to increase with increasing exercise frequency (**Figure 4C**). Serum CRP levels were markedly lower in patients who exercised >3 times/week than in patients who exercised ≤1 time/week (**Figure 4D**). Additionally, we found that exercise duration/week was significantly negatively correlated with PHQ-9 depression scores and high-sensitivity CRP levels and positively correlated with adiponectin and BDNF levels (**Figures 4E–H**). Similarly, exercise duration was negatively correlated with PHQ-9 depression scores and high-sensitivity CRP levels and positively correlated with adiponectin and BDNF levels (**Figures 4I–L**). ROC curve analysis revealed that the AUC values for adiponectin, BDNF, and CRP were greater than 0.75, confirming the excellent diagnostic efficacy of these biomarkers in predicting depression (**Figures 4M–O**).

**Figure 4:**
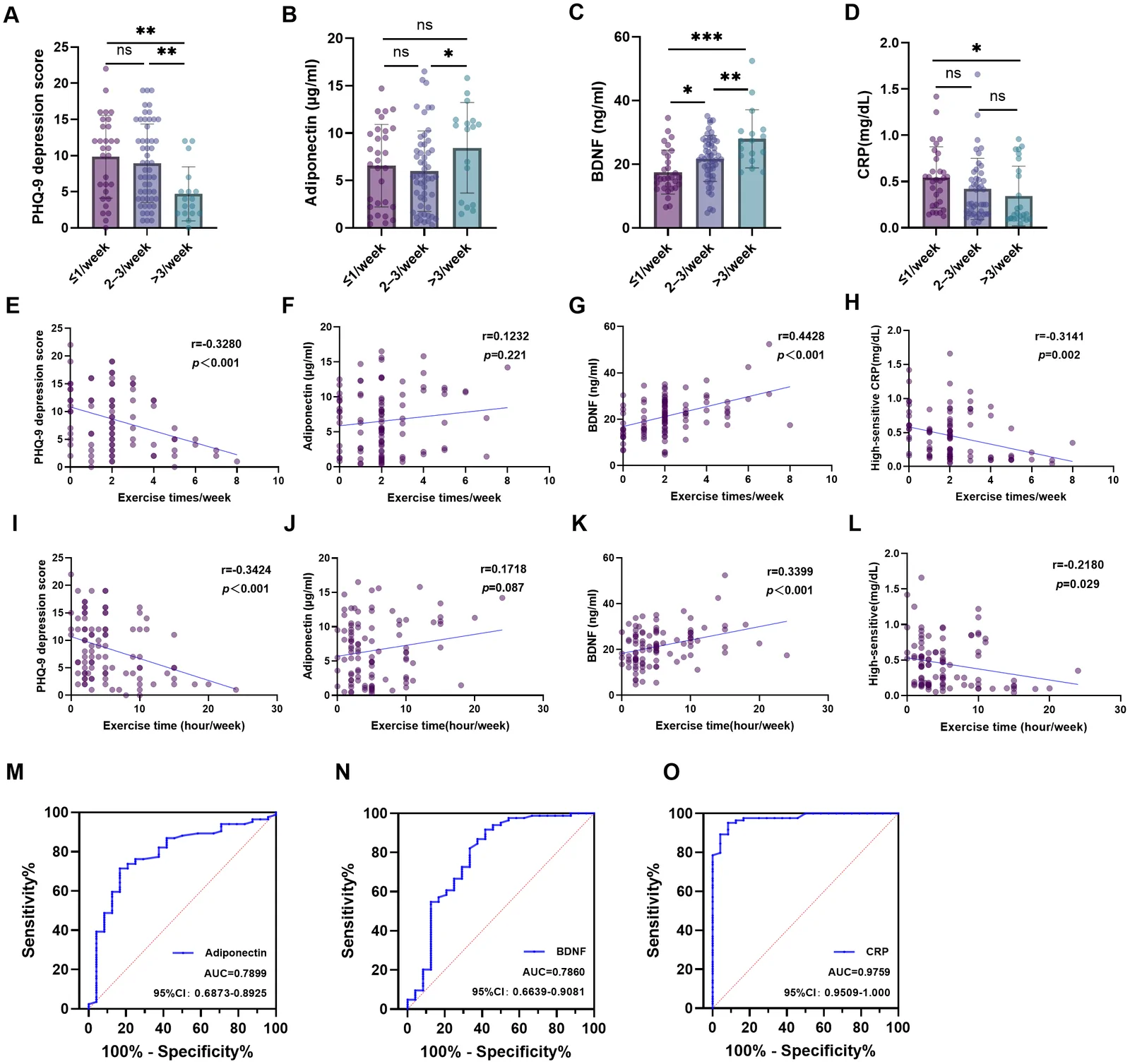
Protective effect of exercise intervention on patients with depression. Effects of exercise frequency on the PHQ-9 depression score **(A)** and adiponectin **(B)**, BDNF **(C)**, and CRP **(D)** levels in patients with depression. Correlation analysis between exercise frequency and PHQ-9 depression scores **(E)**, adiponectin levels **(F)**, BDNF levels **(G)**, and CRP levels **(H)**. Correlation analysis between exercise duration and PHQ-9 depression scores **(I)**, adiponectin levels **(J)**, BDNF levels **(K)**, and CRP levels **(L)**. ROC curves were used to validate the diagnostic efficacy of adiponectin **(M)**, BDNF **(N)**, and CRP **(O)** in patients with depression. *P<0.05, **P<0.01, ***P<0.001, ns P>0.05.

### 3.3 Decreases in adiponectin levels predict depressive-like behavior in mice

We constructed MI and MI+CUMS mouse models to simulate pure HF and HF combined with depression, respectively. On the day of surgery, there was no obvious difference in body weight among the sham, MI, and MI+CUMS groups. By day 7, the body weight of the MI group was much lower than that of the sham group, and the body weight of the MI+CUMS group was significantly lower than that of the MI group (**Figure 5A**). Upon exposure to CUMS, the sucrose preference percentage in the MI+CUMS group was lower than that in the MI group, while there was no significant difference between the MI and sham groups (**Figure 5B**). By day 14, the sucrose preference percentage in the MI group was lower than that in the sham group, and that in the MI+CUMS group was further lower than that in the MI group, indicating that CUMS exacerbated depressive-like behavior in the MI mice. In the tail suspension test, compared with the MI group, the MI+CUMS group exhibited a shorter immobility latency and longer total immobility time (**Figures 5C, D**), which is consistent with the behavioral characteristics of depression. After 14 days of CUMS intervention, compared with the MI group, the MI+CUMS group had higher levels of NT-proBNP and lower levels of adiponectin (**Figures 5E, F**).

**Figure 5:**
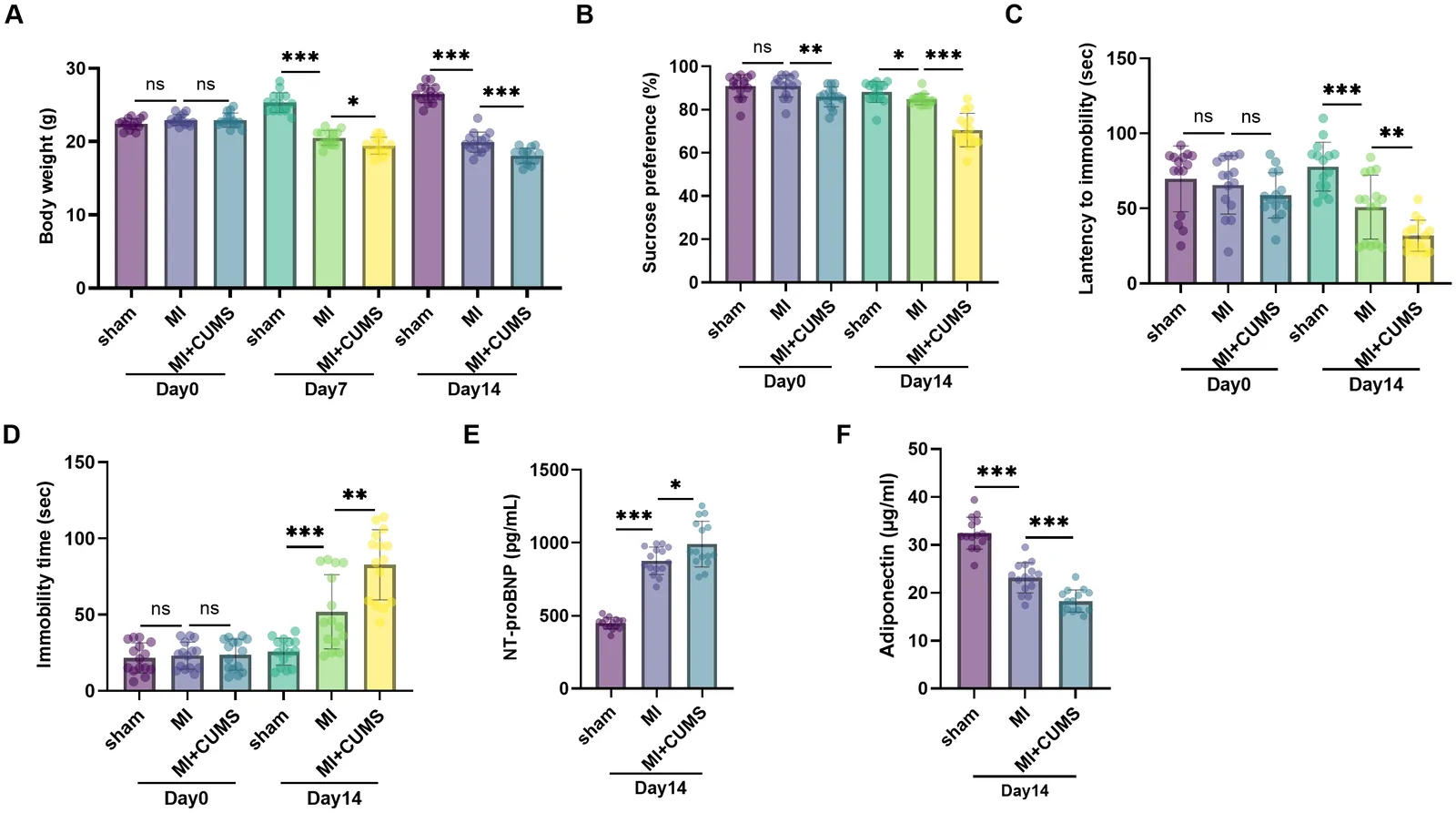
Adiponectin levels are downregulated in heart failure mice with comorbid depression. **(A)** Measurement of weight changes in the MI model and MI+CUMS model mice. **(B)** Sucrose preference test to investigate the effects of the MI model and MI+CUMS model on depressive-like behaviors in mice. Tail suspension test to detect the latency to immobility **(C)** and immobility time **(D)** in the MI group and MI+CUMS group. ELISA detection of serum NT-proBNP **(E)** and adiponectin **(F)** levels in the MI group and MI+CUMS group. *, **, ***, ns means p<0.05, p<0.01, p<0.001, and p>0.05, respectively.

### 3.4 AdipoRon combined with Run intervention effectively improves depressive-like behaviors in mice

To validate the protective effects of adiponectin and exercise on depressive-like behaviors in MI mice, we administered intraperitoneal injections of AdipoRon and a treadmill training regimen to MI+CUMS mice. We found that the combined intervention of AdipoRon and Run significantly increased the body weight of MI+CUMS mice by day 14 (**Figure 6A**), an effect not observed with AdipoRon or Run alone. In the assessment of depressive-like behaviors, compared with a single intervention, the combination of AdipoRon and Run resulted in greater improvement. Although neither AdipoRon nor Run alone improved the percentage of sucrose preference (**Figure 6B**), their combined use markedly enhanced this metric. Further validation through the tail suspension test revealed that, compared with the AdipoRon-alone or Run-alone groups, the combined intervention group exhibited a longer immobility latency (**Figure 6C**) and a shorter total immobility time (**Figure 6D**). Biomarker analysis revealed that compared with the Run-alone group, the AdipoRon+Run group had lower NT-proBNP levels (**Figure 6E**), while adiponectin levels were significantly higher in the AdipoRon+Run group than in the Run group (**Figure 6F**).

**Figure 6:**
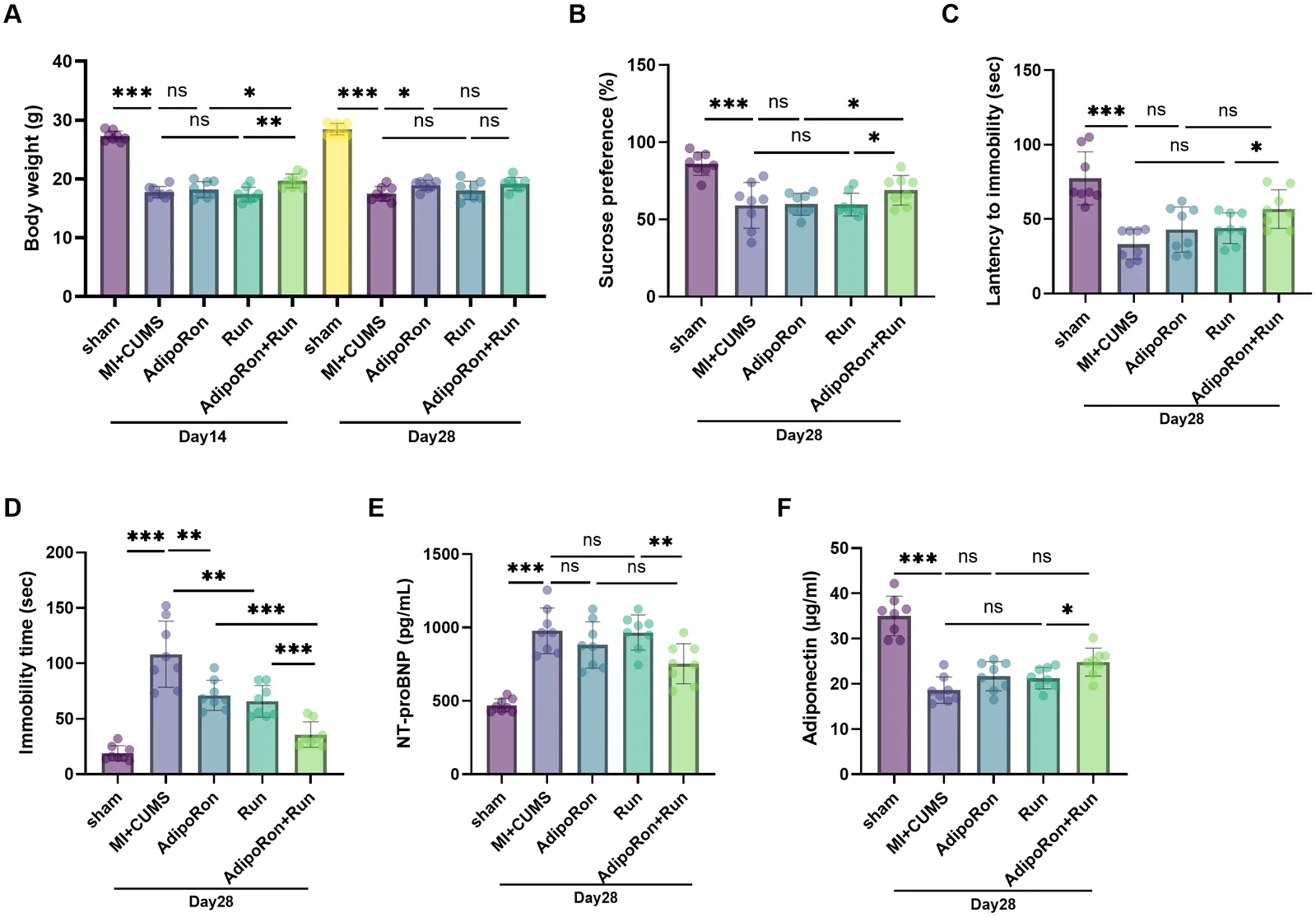
AdipoRon combined with Run intervention effectively improves depressive-like behaviors in mice. **(A)** The effects of AdipoRon and Run intervention on body weight in the MI+CUMS mouse model were examined. **(B)** The sucrose preference test was used to investigate the improvement in depressive-like behaviors in the MI+CUMS mouse model induced by AdipoRon and Run intervention. Effects of AdipoRon and Run intervention on the latency to immobility **(C)** and immobility time **(D)** in the MI+CUMS mouse model were detected by the tail suspension test. ELISA was used to measure the serum levels of NT-proBNP **(E)** and adiponectin **(F)** in the MI+CUMS mouse model following AdipoRon and Run intervention.

## 4 Discussion

In this study, we explored alterations in serum levels of adiponectin, ghrelin, leptin A, and BDNF in CHF patients with/without depression and their clinical associations. Furthermore, we investigated the protective effects of adiponectin and exercise intervention on CHF combined with depression. The results revealed that reduced serum adiponectin levels and exercise activity were associated with increased odds of CHF patients having comorbid depression.

NT-proBNP is an important biomarker released in response to cardiac stress. Elevated levels of NT-proBNP indicate the severity of HF (Shaddy et al., 2025). Wleklik et al. (2025) reported that in HF patients, those with comorbid frailty had significantly higher NT-proBNP levels and worse clinical outcomes. Notably, NT-proBNP is independently associated with increased depression severity in patients with acute myocardial infarction (Ren et al., 2017). Zuzarte et al. (2018) reported that reduced ejection fraction (EF) and NT-proBNP levels were significantly correlated. They further suggested that NT-proBNP can identify depression in patients with HF and reduced EF, and it is also a potential biomarker for evaluating disease progression and mortality. On the other hand, the level of hs-CRP, which is often used as a systemic inflammatory marker, is associated with an increased risk of all-cause mortality in patients with HF. Moreover, hs-CRP levels, depressive symptoms, and NO regulation are codominant risk factors for all-cause mortality in HF patients, even after confounders, such as age, sex, reduced exercise tolerance, and comorbidities, are excluded (Mommersteeg et al., 2016). In this study, we observed that compared with CHF patients, those with CHF and depression had significantly higher NT-proBNP and hs-CRP levels, which were positively correlated with the severity of depression.

Depression is defined as a mood disorder with clinical characteristics and is often accompanied by persistent feelings of sadness, loss of interest, and anhedonia, which are typical psychiatric features of depressive patients (Dudek et al., 2021). Hippocampal synaptic deficits are significant pathological changes associated with depression, and stress can induce dendritic atrophy, reduced synaptic density, and hippocampal synaptic dysfunction (Kallarackal et al., 2013). A controlled study indicated that the 24-hour average adiponectin concentration in female patients with major depressive disorder was 30% lower than that in healthy controls, and adiponectin levels were significantly negatively correlated with the severity of depression and the overall duration of the disease (Cizza et al., 2010). Yang et al. (2019) reported that reduced adiponectin levels at admission in patients with acute ischemic stroke might be associated with poststroke depression (PSD). Diniz et al. (2012) reported that circulating adiponectin levels were decreased in elderly patients with major depressive disorder and that adiponectin levels were positively correlated with Mini-Mental State Examination (MMSE) scores (r = 0.20, p = 0.05). Similarly, among type 2 diabetes patients with mild cognitive impairment, those with both mild cognitive impairment and depressive symptoms exhibited reduced adiponectin levels and elevated hs-CRP and TNF-α levels (Gorska-Ciebiada and Ciebiada, 2024). However, in patients with subsyndromal depression, plasma adiponectin levels were higher than those in nondepressed controls, suggesting that circulating adiponectin may play a role in the compensatory regulation of depressive mood (Jeong et al., 2012). In this study, adiponectin levels and exercise frequency/duration were reduced in CHF patients compared with controls, and adiponectin levels and exercise frequency/duration were further reduced in patients with CHF and depression. To further confirm the effects of alterations in adiponectin in CHF-related depression, CUMS intervention was conducted to induce depression-like symptoms in mice. The experimental results revealed that the MI+CUMS model mice exhibited a significant decrease in sucrose preference percentage (reflecting anhedonia), shortened immobility latency, and prolonged total immobility time in the tail suspension test (reflecting behavioral despair), along with elevated NT-proBNP levels and reduced adiponectin levels.

Exercise intolerance is the primary symptom of HF and is highly significant, as it is linked to reduced quality of life and higher mortality rates (Del Buono et al., 2019). It is widely recognized that physical activity and exercise training significantly influence the biological effects of accelerated aging, thus helping prevent HF (Subramanian et al., 2025). Major society guidelines recommend exercise training for HF patients, which can improve quality of life, alleviate depressive symptoms, and ultimately increase survival and lower the risk of hospitalization (Patti et al., 2021). Our data revealed that compared with CHF patients without depression, CHF patients with depression had reduced exercise frequency and exercise duration, suggesting that a decrease in exercise frequency/duration is a potential risk factor for depression in CHF patients. However, it is important to note that MI or CHF can independently cause fatigue, reduced locomotor activity, and sickness behavior (e.g., lethargy, anhedonia-like states) (Hill et al., 2005; Kang et al., 2025). Therefore, the observed behavioral changes may not represent pure depressive-like behavior but rather a composite phenotype of HF-related malaise and comorbid depression. This overlap limits the specificity of the behavioral assays in the MI+CUMS model. The MI+CUMS model does not fully elucidate depression-specific mechanisms—HF induced by MI is a chronic systemic disease accompanied by fatigue, reduced physical activity, systemic inflammation, and metabolic dysregulation, all of which can produce behavioral changes that overlap with classic depressive-like symptoms. Consequently, our findings should be interpreted as reflecting the comorbid condition of HF with depressive features rather than pure major depressive disorder. Similarly, the observed changes in adiponectin and CRP levels may represent systemic alterations common to both HF and depression rather than pathways uniquely linked to depression alone. Future studies should include additional behavioral tests (e.g., nest building, burrowing) or physiological markers to better dissociate HF-induced sickness behavior from depressive-like symptoms. Moreover, studies using depression models without cardiovascular comorbidities are needed to determine whether the interaction between adiponectin and exercise is specific to depression or generalizable to negative behavioral symptoms associated with other chronic diseases.

Furthermore, it is necessary to mention that in CHF, a paradoxical relationship between adiponectin levels and disease outcome has been described. Although adiponectin generally exerts cardioprotective effects (e.g., improving insulin sensitivity and reducing inflammation) (Kazemi et al., 2025), elevated circulating adiponectin levels in CHF patients have been paradoxically associated with advanced disease severity, cardiac cachexia, and poor prognosis (Araújo et al., 2009; Celik and Yaman, 2010; Mado et al., 2021). This phenomenon, known as the ‘adiponectin paradox’, may reflect a compensatory response to cardiac dysfunction or the development of adiponectin resistance. In this study, we observed that low adiponectin levels were associated with the occurrence of depression. Patients with CHF and high-frequency exercise had increased adiponectin levels. These findings may contradict the adiponectin paradox. However, it should be emphasized that the adiponectin paradox primarily pertains to the relationship between elevated adiponectin levels and worse cardiovascular outcomes (e.g., mortality and cachexia), whereas our study focused on depression and its modulation by exercise. Moreover, the adiponectin paradox has been reported predominantly in patients with advanced, cachectic CHF, whereas our cohort consisted of noncachectic CHF patients with depression. Further studies are needed to clarify how the adiponectin paradox interacts with depressive symptoms and exercise interventions.

Currently, selective serotonin reuptake inhibitors (SSRIs) and serotonin-norepinephrine reuptake inhibitors (SNRIs) remain the first-line medications for treating depression (Kessler et al., 2018). However, a major clinical challenge is that a substantial proportion of patients (approximately 50%) do not achieve an adequate response to SSRIs, and many experience only partial remission (Caspersen et al., 1985). This issue is particularly relevant for patients with comorbid conditions, such as CHF, where metabolic and inflammatory dysregulation may further compromise treatment efficacy. Therefore, our research has emphasized the importance of identifying specific patient subtypes on the basis of metabolic alterations (e.g., adiponectin, BDNF, and inflammatory markers) to guide more targeted and effective interventions. In this context, physical exercise has emerged as an alternative therapy for depression because of its safety and accessibility. When physical activity is structured, planned, and aimed at improving or maintaining physical health, it is referred to as exercise (Rush et al., 2006). Regular exercise can significantly influence emotional states. On the one hand, it enhances self-efficacy, intrinsic motivation, and energy reserves while improving psychosocial functioning. On the other hand, regular exercise improves neurobiological effects, such as increased hippocampal neurogenesis, monoamine neurotransmitter transmission, and synaptic growth processes (Dremencov et al., 2017). Liu et al. (2024) reported that regular physical exercise (e.g., a four-week running regimen) improved depressive-like behaviors in mice. The mechanism is closely related to elevated adiponectin levels and upregulated AdipoR1 expression in the hippocampus. The adiponectin/AdipoR1 pathway effectively suppresses hippocampal neuroinflammation by regulating the balance of microglial M1/M2 polarization. Adiponectin can cross the blood–brain barrier by binding to adiponectin receptors, directly modulating synaptic plasticity (Schön et al., 2019). Moreover, exercise significantly increases BDNF levels in the hippocampus and other brain regions (Voss et al., 2013). As a member of the neurotrophin family, BDNF is synthesized in the brain and plays a central role in neuronal survival, growth, maintenance, and learning–memory functions, serving as a key regulator of activity-dependent neuronal plasticity (Albini et al., 2023). Studies have indicated that BDNF has a decisive effect on hippocampal neurogenesis and the pathological development of depression, improving depressive-like behaviors by promoting neuronal survival, synaptic function, and optimization (Einat et al., 2003). Yang et al. (2023) demonstrated that enhanced adiponectin activity and upregulated hippocampal AdipoR1 expression activate downstream BDNF signaling pathways, synergistically producing antidepressant effects. In our clinical study, the high-frequency exercise group showed significant increases in serum adiponectin and BDNF levels and marked improvements in PHQ-9 depression scores. Correlation analysis revealed that exercise frequency and duration were positively associated with serum BDNF levels. Animal model validation revealed that compared with single interventions, the combination of adiponectin receptor agonists and exercise intervention resulted in significantly superior antidepressant efficacy, providing scientific evidence for the use of exercise–drug combination therapies in the treatment of depression.

When interpreting our research results, one should carefully consider the potential confounding effects of diabetes and systemic inflammation on depression. Previous studies have shown that the hs-CRP level in patients with type 2 diabetes is positively correlated with depression and anxiety (Yang et al., 2020), suggesting that hyperglycemia may induce or exacerbate depressive and anxious states through the inflammatory pathway. Moreover, complications related to diabetes have been proven to significantly increase the risk of depression and anxiety (HR = 1.77, 95% CI: 1.73–1.80) (Horsbøl et al., 2024). More importantly, diabetes is a known risk factor for HF, and certain hypoglycemic drugs, including thiazolidinediones and sulfonylureas, are associated with an increased risk of HF (Nissen and Wolski, 2007; Han et al., 2022), further complicating the interactions between these diseases. All of these factors may have confounded the observed association between adiponectin levels and depression in our analysis.

### 4.1 Limitations

This study has several limitations. First, the interventions of AdipoRon treatment and exercise were applied only to the MI + CUMS group; other experimental groups (e.g., MI alone or Sham alone) did not receive these interventions. Therefore, the specificity of the observed beneficial effects on comorbid conditions remains unclear. Second, a Sham + CUMS group was not included in the experimental design. Thus, determining whether the depressive-like behaviors induced by CUMS in MI mice represent an additive or potentiating effect of the comorbidity or simply the general effect of CUMS independent of MI might be difficult. Third, no histopathological examinations of brain tissue (such as neuronal apoptosis, glial cell activation, or inflammatory cytokine infiltration) were conducted in the mice, making it impossible to verify whether the depressive phenotype was accompanied by neuroinflammation or neuronal damage. Fourth, the control group received no injection of vehicle, which may introduce bias, as the stress of handling and injection could independently influence behavioral outcomes. Future studies should include a vehicle-injected control group to avoid potential bias. Fifth, each intervention group included only 8 mice, and the small sample size may have led to insufficient statistical power, an increased risk of random errors, and compromised the reproducibility of the results. Finally, in our clinical survey, we reported that higher self-reported exercise frequency was associated with lower depression scores. However, the observational design precludes the determination of causality. Specifically, patients with more severe HF and greater depressive symptoms are likely to have reduced exercise capacity because of dyspnea, fatigue, and a lack of motivation. Thus, lower physical activity levels could be a consequence rather than a cause of depression (i.e., reverse causation). Our study cannot distinguish whether exercise protects against depression or whether less depressed individuals are simply more able to exercise. In conclusion, we demonstrated that adiponectin receptor agonists combined with exercise intervention effectively improved depressive-like behaviors in mice and modulated related biomarker levels, providing a new direction for the comprehensive clinical management of CHF with comorbid depression.

## 5 Conclusion

While our sensitivity analyses (forest plot) confirmed the robustness of these associations across various high-risk subgroups, such robustness does not imply causality. We cannot exclude the possibility of reverse causation (e.g., depressive symptoms leading to reduced physical activity) or residual confounding from unmeasured variables (e.g., medication adherence and nutritional status). In summary, our observational clinical data show that reduced serum adiponectin levels are associated with a higher incidence of depression in patients with CHF. In a mouse model of CHF with comorbid depression, activation of the adiponectin/AdipoR1 pathway combined with exercise intervention ameliorated depressive-like behaviors, suggesting a potential protective effect. Together, these findings provide preliminary scientific evidence supporting the further investigation of adiponectin combined with exercise intervention as a possible component of comprehensive treatment strategies for CHF patients with comorbid depression, particularly in the context of brain-heart axis regulation. However, owing to the observational nature of the clinical data, causal relationships cannot be established, and future prospective studies are needed to confirm these findings.

## Data Availability

The original contributions presented in the study are included in the article/supplementary material. Further inquiries can be directed to the corresponding author.
